# Literary Notes

**Published:** 1897-07

**Authors:** 


### Literary Notes.

Addendum.—Mr. Lawson Tait sends the following foot-note to his article published in the June edition of this journal, page 805, second line from the bottom, placing an asterisk after the word "cysts":

"Dr. Howard Kelly, of Baltimore, has lately stumbled on this fact and has published it as a great discovery. It has been indicated at length in everything I have written on the subject for a quarter of a century. The professor at Johns Hopkins University is, as usual, a long way behind the times." L. T.

THE American Journal of Insanity, heretofore published at 34 Washington street, Chicago, will be transferred to Baltimore, July 1, 1897, and will be published by the Johns Hopkins Press. The editorial control will be in the hands of a committee of the American Medico-Psychological Association, consisting of Dr. Henry M. Hurd and Dr. E. N. Brush, of Baltimore; Dr. G. Alder Blumer, of Utica, N. Y., and Dr. J. Montgomery Mosher, of Albany, N. Y.

All communications for the Journal should be addressed to Dr. Henry M. Hurd, care of the Johns Hopkins Hospital, Baltimore, or to any of the editors. All exchanges and business communications should be addressed to the Johns Hopkins Press, Baltimore.

---

*Greater Buffalo* is the name of a monthly magazine devoted to promoting the prosperity of the power city of America, lately established at Buffalo. The first number is a well-printed and handsomely illustrated paper that promises a successful career. It is published by the Greater Buffalo Publishing Company from its office, 720 Real Estate Exchange.

---

MESSRS. E. B. Treat & Company have removed from 5 Cooper Union to 241–243 West Twenty-third street, New York. William H. and Edwin C. Treat, sons of E. B. Treat, who have been associated with the father in business for several years, have been admitted to partnership under the above firm name.

